# Retraction: Targeted plasmalogen supplementation: effects on blood plasmalogens, oxidative stress biomarkers, cognition, and mobility in cognitively impaired persons

**DOI:** 10.3389/fcell.2026.1964604

**Published:** 2026-08-13

**Authors:** Frontiers Editorial Office

The journal retracts the 06 July 2022 article cited above.

Following publication, concerns were raised regarding the ethical procedures and the scientific validity of the article.

An investigation was conducted in accordance with Frontiers’ policies, and it was found that the complaints were valid and that the article does not meet the standards of editorial and scientific soundness for Frontiers in Cell and Developmental Biology. It was confirmed that, contrary to the statement in the article, the study was not approved by Sterling IRB. Lack of appropriate ethical approval is a breach of Frontiers’ guidelines and those of the Committee on Publication Ethics. As such, this article is being retracted.

This retraction was approved by the Chief Editors of Frontiers in Cell and Developmental Biology and the Chief Executive Editor of Frontiers. The authors have not responded to correspondence regarding this retraction.

Frontiers would like to thank the concerned reader who contacted us regarding the published article.

